# Influence of stimulus manipulation on conscious awareness of emotional facial expressions in the match-to-sample paradigm

**DOI:** 10.1038/s41598-023-47995-9

**Published:** 2023-11-25

**Authors:** Wataru Sato, Sakiko Yoshikawa

**Affiliations:** 1 Psychological Process Research Team, Guardian Robot Project, RIKEN, 2-2-2 Hikaridai, Seika-cho, Soraku-gun, Kyoto, 619-0288 Japan; 2 Field Science Education and Research Center, Kyoto University, Oiwake-cho, Kitashirakawa, Sakyo, Kyoto, 606-8502 Japan; 3 Faculty of the Arts, Kyoto University of the Arts, 2-116 Uryuyama, Kitashirakawa, Sakyo, Kyoto, 606-8501 Japan

**Keywords:** Human behaviour, Emotion

## Abstract

The conscious perception of emotional facial expressions plays an indispensable role in social interaction. However, previous psychological studies have reported inconsistent findings regarding whether conscious awareness is greater for emotional expressions than for neutral expressions. Furthermore, whether this phenomenon is attributable to emotional or visual factors remains unknown. To investigate these issues, we conducted five psychological experiments to test the conscious perception of emotional and neutral facial expressions using the match-to-sample paradigm. Facial stimuli were momentarily presented in the peripheral visual fields while participants read simultaneously presented letters in the central visual fields. The participants selected a perceived face from nine samples. The results of all experiments demonstrated that emotional expressions were more accurately identified than neutral expressions. Furthermore, Experiment 4 showed that angry expressions were identified more accurately than anti-angry expressions, which expressed neutral emotions with comparable physical changes to angry expressions. Experiment 5, testing the interaction between emotional expression and face direction, showed that angry expressions looking toward participants were more accurately identified than those looking away from participants, even though they were physically identical. These results suggest that the conscious awareness of emotional facial expressions is enhanced by their emotional significance.

## Introduction

The prioritized perception of emotional stimuli, including the emotional facial expressions of conspecifics, is one of the most important adaptive mechanisms^1,2^. Appropriate conscious recognition of the emotional expressions of others allows us to understand their emotional states, and thus promotes the creation and maintenance of social relationships^3^, as conscious processing is more flexible than automatic, unconscious responses^4–7^.

Numerous psychology studies have demonstrated that the conscious perception of emotional facial expressions is more efficient than that of emotionally neutral expressions using several different paradigms including backward masking^8–11^, attentional blink^12–17^, and continuous flash suppression^18–21^. For instance, Esteves and Öhman^8^ presented photographs of angry, happy, and neutral facial expressions for short durations (ranging from 20 to 300 ms), with neutral faces as the backward masking stimuli. Participants were asked to recognize the presented facial expressions. The threshold for the conscious detection of happy facial expressions was shorter than that of neutral expressions. Milders et al.^12^ used the attentional blink paradigm and presented photographs of fearful or neutral expressions as the second target face in 50% of trials during the rapid serial visual presentation of scrambled faces; each image was presented for 80 ms and was immediately masked by the next image. Participants reported whether they had seen a second face by pressing a key. The conscious detection of fearful faces was more accurate than that of neutral faces. Yang et al.^18^ used the continuous flash suppression paradigm and presented fearful or neutral faces to one eye while making the stimuli invisible for relatively long periods by presenting dynamic noise patch suppressors to the other eye. The fearful faces became visible earlier than the neutral faces. These psychological data are consistent with neurophysiological^22^ and neuroimaging^23^ evidence that activity in the visual cortices is enhanced during the processing of emotional facial expressions compared with neutral expressions. For example, a previous study recorded electroencephalography during the observation of photographs of fearful, happy, and neutral expressions and found that the amplitudes of negative deflections at about 270 ms, covering a broad visual area, were stronger in response to fearful and happy expressions than to neutral expressions^22^. Other neuroscientific studies indicated that the amygdala conducts emotional processing for facial expressions before conscious awareness^24,25^ via the subcortical visual pathway^26^, and modulates activity in the visual cortices during the conscious processing of facial expressions^27,28^. Taken together, these data suggest that the conscious awareness of emotional facial expressions is enhanced compared with neutral expressions, due to the importance of emotional information.

However, this idea remains controversial because several studies have reported inconsistent findings^29^. For example, Miders and Sahraie^10^ investigated the discrimination of photographs of angry, fearful, happy, and neutral faces in the backward masking paradigm and found that relative to neutral expressions, performance was superior for happy but not angry or fearful expressions. Sun et al.^17^ tested the conscious detection of fearful, happy, and neutral expressions using the attentional blink paradigm and found that detection performance was better for happy, but not fearful, expressions than neutral expressions. Hedger et al.^11^ tested the conscious awareness of emotional (angry, fearful, and happy) and neutral expressions using the continuous flash suppression paradigm and found that the conscious perception of emotional expressions was not superior to that of neutral expressions. These discrepancies may be related to specific limitations of the experimental paradigms. For example, in the backward masking and attentional blink paradigms, target stimulus presentation is brief with subsequent masking images, which may induce difficulty to produce clear effects^30^. Psychological and neuroimaging findings suggest that the unconscious processing during the continuous flash suppression may be restricted to low-level visual features of the stimuli^31^. Previous lesion studies have shown that blindsight patients can infer emotions in facial expressions^32–34^ and the presence of faces^34^ better than chance in the absence of conscious awareness of faces; thus, emotion recognition and simple alternative-forced choice tasks may reflect unconscious processing, at least partially. Hence, more investigation using different paradigms is warranted^29^. We hypothesized that it would be possible to demonstrate that the conscious awareness of emotional facial expressions is enhanced compared with that of neutral facial expressions using a different paradigm from those used in the previous studies.

Furthermore, whether the enhanced conscious awareness of emotional versus neutral facial expressions is due to emotional or visual factors remains unclear. Emotional and neutral facial expressions not only have different emotional significance but also different physical features (e.g., oblique eyebrows in angry expressions versus horizontal eyebrows in neutral expressions). Some studies have demonstrated that several visual features, such as oblique lines and curves, are detected more efficiently than other features, such as horizontal lines^35,36^. Consistent with these data, some previous studies have shown that the enhanced conscious perception of emotional versus neutral facial expressions could be driven by visual factors rather than emotional factors^11,20,21^. For example, Gray et al.^21^ used the continuous flash suppression paradigm and found that while photographs of emotional facial expressions were more rapidly detected than those of neutral expressions; this was also the case for inverted and negated versions of emotional facial expression photos, which had less emotional impact. This issue has been explored using the visual search paradigm, which assesses attention according to reaction time measures but does not necessarily measure conscious awareness^37^. Several studies compared the detection speed of photographs of normal angry and happy facial expressions with those of the corresponding anti-expressions in a crowd of neutral faces^38–46^. Anti-expressions are artificial stimuli with a degree of visual change equivalent to that of emotional facial expressions; however, the anti-expressions were recognized as neutral expressions during free categorical labeling^47^. The results of such studies consistently demonstrated that the reaction times for detecting normal emotional expressions were shorter than those for their corresponding anti-expressions. These findings suggest that emotional significance contributes to the rapid detection of emotional expressions. Hence, we hypothesized that emotional facial expressions could stimulate enhanced conscious awareness compared with neutral expressions due to their emotional significance, irrespective of their visual features.

To test these hypotheses, we examined the conscious perception of photographs of emotional and neutral facial expressions using the match-to-sample paradigm^48,49^. In each trial in this paradigm, previous researchers first presented a letter in the central field. Subsequently, the participants were shown a schematic facial expression in the left or right peripheral visual field rapidly (e.g. 100 ms^49^), followed by a test panel with nine facial expressions. The participants were requested to first report the letter and then to select the face from the test panel. The first letter-report task was introduced to ensure fixation^48^; this gave the trials a dual-task structure, which made the perception of facial expressions more difficult^49^. The secondary fixation task was originally developed to test hemispheric functional asymmetries^50,51^ and has been used in numerous neuropsychological studies^52^. Researchers confirmed that active fixation tasks suppressed micro-saccades more than passive ones^53^. A previous study that analyzed the differences between negative, positive, and neutral emotions found that the accuracy in identifying negative and positive facial expressions was higher than that for neutral expressions, and that the accuracy was greater for negative expressions than for positive expressions^49^. These results suggest that the conscious perception of emotional facial expressions is more accurate than that for neutral expressions, specifically for negative emotions. However, the evidence is inconclusive because the researchers did not statistically clarify the differences between the emotional conditions. Furthermore, as schematic faces are artificial and simplified depictions of human faces, they may lack ecological validity compared with photographs of human faces^54^. Previous studies have reported that psychological^55^ and neural^56^ processing of pictorial facial expressions was weaker than that of photographs. Hence, an investigation using photographs of facial expressions was warranted. We reasoned that the match-to-sample paradigm offers several advantages in the investigation of the conscious awareness of emotional facial expressions. First, stimuli can be presented for a relatively long duration without masking stimuli or suppressors. Second, the visual factors of the expression stimulus can be uniquely controlled (e.g., Experiment 5). Finally, consciously perceived images of facial expressions can be assessed in detail.

**Figure 1:**
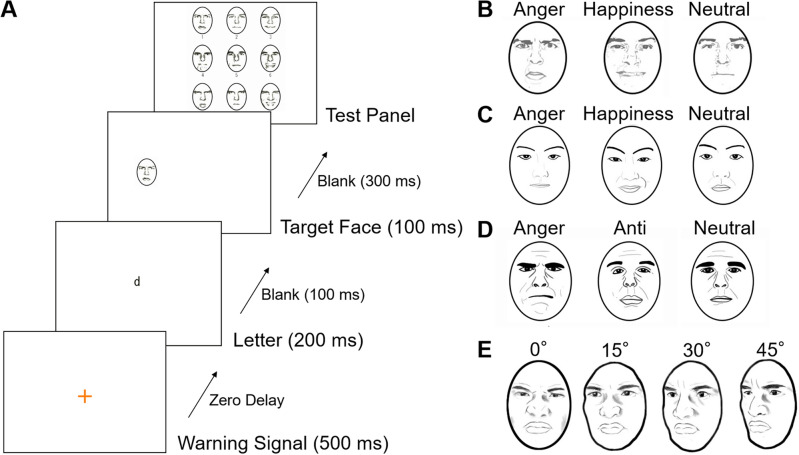
Illustrations of the match-to-sample paradigm (**A**) and stimuli in Experiments 1 ((**B**) Caucasian male’s angry, happy, and neutral expressions), 3 ((**C**) Japanese female’s angry, happy, and neutral expressions), 4 ((**D**) normal-angry, anti-angry, and neutral expressions), and 5 ((**E**) angry expressions at face angles of 0°, 15°, 30°, and 45°). Actual stimuli were photographic faces. The images in the figure were drawn by the authors.

We conducted five experiments using the match-to-sample paradigm with photographic faces (Fig. 1A), and statistically compared the effect of emotion. In Experiments 1–3 (Fig. 1B and C), we tried to replicate previous findings regarding the enhanced conscious perception of emotional versus neutral facial expressions using various types of facial expressions showing angry, happy, and neutral emotions. The test panels contained nine faces with three individuals showing three different expressions, one of which was the target face. To assess the conscious perception of facial expressions but not unconscious emotional information processing^57^, the participants were requested to identify the consciously perceived images without guessing. Because previous studies testing the detection of photographs of facial expressions using the visual search paradigm have shown confounding effects of physical artifacts, such as dark areas^58,59^, we aimed to replicate the findings using different facial stimuli, including Caucasian males (Experiment 1), Japanese males (Experiment 2), and Japanese females (Experiment 3). Previous studies have reported physical differences (e.g. color) between Caucasian and Asian, and male and female, faces^60^. Based on the aforementioned studies that tested conscious awareness of emotional expressions^8–21^, we predicted that identification accuracy would be higher for angry and happy expressions compared with neutral expressions. Because the previous studies reported inconsistent findings regarding valence differences^8–14,16–18,20,21^, we examined the difference between angry and happy expressions without making specific predictions. We also exploratorily analyzed the effect of the visual field because previous studies using the match-to-sample paradigm with schematic faces have reported inconsistent findings^48,49^.

Then we tested the influence of emotional versus visual factors in Experiments 4 and 5. In Experiment 4, we used normal-angry, anti-angry, and neutral facial expressions as stimuli (Fig. 1D). As described above, anti-expressions conveyed neutral emotions but had visual feature changes comparable with those for emotional facial expressions^47^. A recent lesion study using the visual search paradigm showed that the rapid detection of normal-angry expressions compared with anti-angry expressions was impaired after amygdala damage^44^. Based on this finding, together with ample evidence suggesting amygdala involvement in enhanced conscious awareness of emotional facial expressions described above, we predicted that normal-angry expressions would be more accurately identified than anti-expressions. In Experiment 5, we manipulated the face directions of angry, happy, and neutral expressions in the unilateral visual fields (Fig. 1E); this allowed us to alter the emotional significance of the facial expression for the observer without affecting the physical features of the expression. A previous neuroimaging study similarly manipulated the directions of angry and neutral expressions in the unilateral visual fields and found that angry expressions looking toward the participants elicited stronger amygdala activation than angry expressions looking away from the participants^61^. Based on this finding, together with the evidence of amygdala involvement, we predicted that angry expressions looking toward participants would be more accurately identified than those looking away from the participants.

## Results

### Experiments 1

Experiment 1 was conducted to replicate the advantage of emotional versus neutral facial expressions in the match-to-sample paradigm^49^ using photographic stimuli. The target faces were grayscale photographs of Caucasian males, chosen from a standard set^62^ (Fig. 1B). We compared emotional expressions with two emotional valences (angry as negative and happy as positive) with neutral expressions.

**Figure 2:**
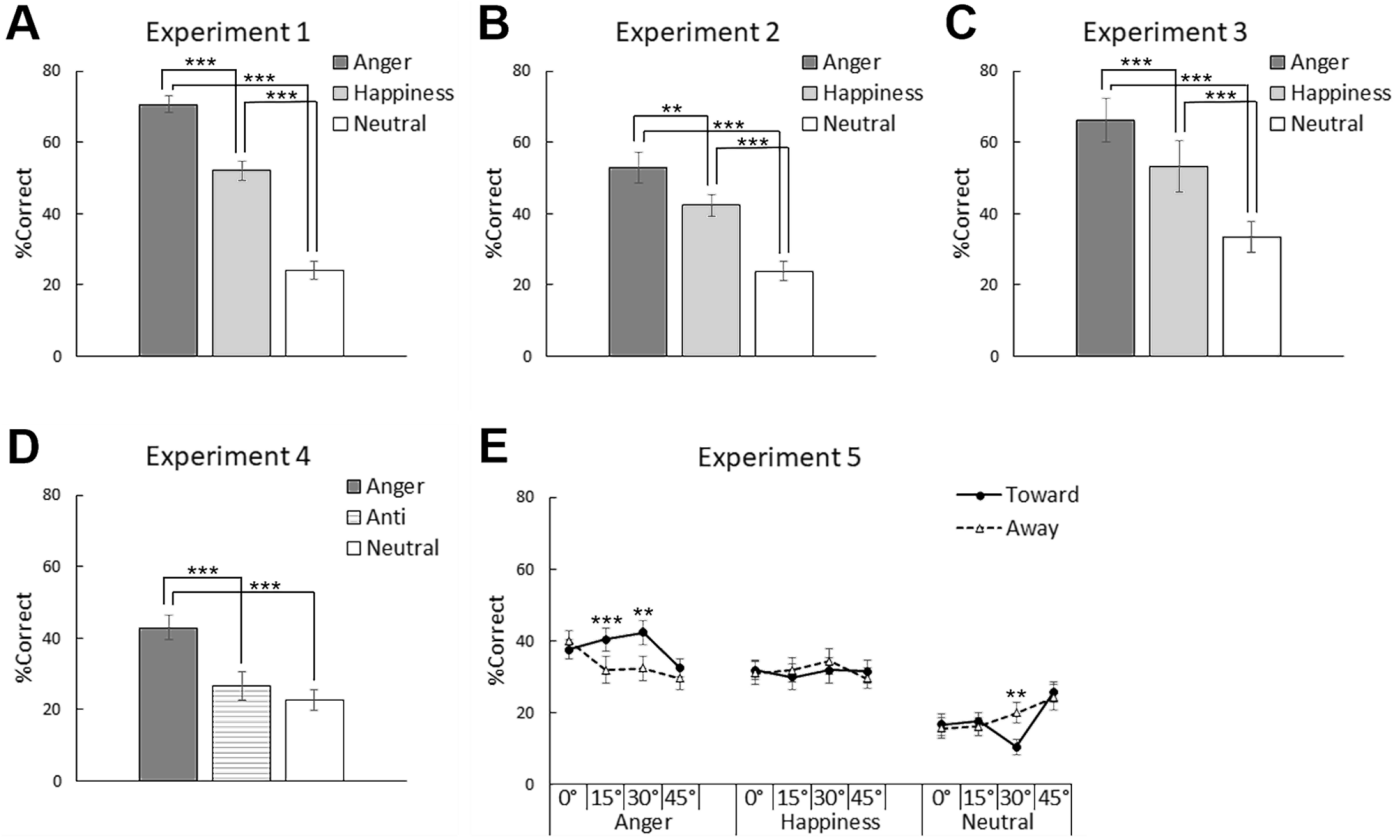
Mean (with standard error) identification accuracy (%) in Experiments 1 (**A**), 2 (**B**). 3 (**C**), 4 (**D**), and 5 (**E**). Asterisks indicate significant simple effects of emotion in Experiments 1–4 and those of face direction in Experiment 5. ***p < 0.001; **p < 0.005; *p < 0.05.

We performed a two-way repeated-measures analysis of variance (ANOVA) for the percentage of trials in which the target was correctly identified (Fig. 2A), with emotion (anger, happiness, and neutral) and visual field (left and right) as within-subjects factors. The results showed a significant main effect of emotion (*F*(2,34) = 158.7, *p* = 0.000, *η*^2^_*p*_ = 0.90). The main effect of visual field and the interaction between emotion and visual field were not significant (*F* < 2.2, *p* > 0.128, *η*^2^_*p*_ < 0.12). Follow-up multiple comparisons for the main effect of emotion revealed that the percentage of correct identification of angry and happy expressions was significantly higher than that for neutral expressions and that the identification of angry expressions was significantly better than that for happy expressions (*t*(34) > 7.1, *p* < 0.001).

### Experiments 2

To examine the reliability of the findings, we conducted Experiments 2 and 3, in which the target faces were replaced with color photographs of Japanese males and females, respectively (Fig. 1C).

As in Experiment 1, a two-way repeated-measures ANOVA of the percentage of correctly identified faces in Experiment 2 (Fig. 2B) revealed a significant main effect of emotion (*F*(2,24) = 32.2, *p* = 0.000, *η*^2^_*p*_ = 0.76). The main effect of visual field showed a nonsignificant trend (*F*(1,12) = 4.7, *p* = 0.051, *η*^2^_*p*_ = 0.28), indicating a left visual field advantage. The interaction between emotion and visual field was not significant (*F*(2,24) = 0.5, *p* = 0.624, *η*^2^_*p*_ = 0.04). Multiple comparisons for the main effect of emotion revealed that the detection of angry and happy expressions was significantly better than that for neutral expressions, and that for angry expressions was significantly better than for happy expressions (*t*(24) > 3.0, *p* < 0.005).

### Experiments 3

Likewise, a two-way repeated-measures ANOVA of Experiment 3 (Fig. 2C) revealed a significant main effect of emotion (*F*(2,22) = 45.4, *p* = 0.000, *η*^2^_*p*_ = 0.80). The main effect of visual field and the emotion × visual field interaction were not significant (*F* < 0.9, *p* > 0.372, *η*^2^_*p*_ < 0.07). Multiple comparisons for the main effect of emotion showed that the detection performance of anger, happiness, and neutral expressions significantly differed in the same order as in Experiments 1 and 2 (*t*(22) > 4.3, *p* < 0.001).

### Experiment 4

To examine the effect of visual factors, we conducted Experiment 4 using normal-angry, anti-angry, and neutral expressions (Fig. 1D). The anti-angry expressions had a comparable degree of distinctiveness with respect to the normal-angry expressions but with the emotional load effectively filtered out^47^. If perceptual enhancement occurred as the result of emotional processing, the performance in a match-to-sample task for artificial expressions might not be as good as that for faces with negative expressions, despite the equalized distinctiveness of visual features.

We analyzed the percent correct identification (Fig. 2D) using a two-way repeated-measures ANOVA with emotion (normal-anger, anti-anger, and neutral) and visual field (left and right) as within-subjects factors. The results revealed a significant main effect of emotion (*F*(2,34) = 15.7, *p* = 0.000, *η*^2^_*p*_ = 0.48). The main effect of the visual field and the emotion × visual field interaction were not significant (*F* < 1.5, *p* > 0.250, *η*^2^_*p*_ < 0.08). Follow-up multiple comparisons revealed that the normal-angry expressions were identified more accurately than both the anti-angry and neutral expressions (*t*(34) > 4.2, *p* < 0.001). There were no significant differences between the anti-angry and neutral expressions (*t*(34) = 1.1, *p* = 0.302).

### Experiment 5

To further test the influence of emotional and visual factors, we investigated the perception of angry, happy, and neutral facial expressions in which the photographs of individuals were looking toward or away from the participants at angles of 0°, 15°, 30°, and 45° (Fig. 1E). Although the visual features of the stimuli were identical between the toward and away face direction conditions, their emotional impact could differ. The preliminary experiments with naïve participants indicated that faces looking toward the participants at angles of 15° and 30° produced the impression of eye contact in the current experimental environment.

We analyzed the percent correct identification (Fig. 2E) using a three-way repeated-measures ANOVA with emotion (anger, happiness, and neutral), face direction (toward and away), and face angle (0°, 15°, 30°, and 45°) as within-subjects factors. As in the above experiment, the results showed a significant main effect of emotion (*F*(2,68) = 53.9, *p* > 0.205, *η*^2^_*p*_ = 0.51). Additionally, two-way interactions between emotion and face direction (*F*(2,68) = 6.0, *p* = 0.004, *η*^2^_*p*_ = 0.10) and between emotion and face angle (*F*(6,204) = 4.4, *p* = 0.000, *η*^2^_*p*_ = 0.12), and the three-way interaction of emotion, face direction, and face angle (*F*(6,204) = 2.7, *p* = 0.020, *η*^2^_*p*_ = 0.05) were significant. Other main effects or interactions were not significant (*F* < 1.6, *p* > 0.250, *η*^2^_*p*_ < 0.02). To confirm the overall emotion effect, follow-up multiple comparison tests were conducted for the main effect of emotion. Identification accuracy was significantly higher for angry and happy expressions than for neutral expressions, as well as for angry expressions than for happy expressions (*t*(68) > 2.8, *p* < 0.01).

For the interactions, follow-up analyses of simple-simple main effects of face direction (toward versus away) were analyzed based on our interest. The results showed that angry faces looking toward the participants were detected more efficiently than angry faces looking away from the participants at angles of 15° and 30° (*F*(1,408) > 8.3, *p* < 0.005). Unexpectedly, neutral faces looking away from the participants were significantly more easily detected than neutral expressions looking toward the participants at 30° (*F*(1,408) = 7.2, *p* = 0.008). There were no other significant differences between the toward and away face direction conditions (*F* < 1.4, *p* > 0.244).

## Discussion

Consistent with our predictions, the results of all experiments demonstrated that emotional expressions were more accurately identified than neutral expressions. Specifically, the results of Experiment 1, which used photographs of Caucasian males in a match-to-sample paradigm, showed that the identification accuracy of emotional (both angry and happy) expressions was higher than that of neutral expressions. The results of Experiments 2 and 3 replicated those of Experiment 1 using photographs of Japanese males and females. Experiments 4 and 5 similarly showed an identification advantage for emotional versus neutral expressions. The experimental redundancy observed in these experiments suggests that the perceptual enhancement of emotional versus neutral expressions is a reliable psychological phenomenon. Because the physical characteristics of the stimuli clearly differed among our experiments, the results are not likely to be attributable to stimulus artifacts^58,59^. In addition, because the stimuli differed in race and gender, the findings appear to reflect universal psychological activity. Our results are largely consistent with previous studies reporting enhanced conscious perception of emotional expressions relative to neutral expressions using different paradigms, including backward masking^8–11^, attentional blink^12–17^, and continuous flash suppression^18–21^. However, there was some degree of inconsistency in the results, possibly due to paradigm-specific limitations; thus, additional studies using different paradigms are needed. We speculate that certain methodological advantages of the match-to-sample paradigm, including stimulus presentation within a specific amount of time and detailed assessment of conscious perception using sample matching, provide more reliable results. Our results are also compatible with the findings of a previous study that used the match-to-sample paradigm^49^. However, that study did not statistically test the differences in identification accuracy between emotion conditions, and used only schematic drawings of faces as stimuli. Our results extend these findings and indicate that the conscious perception of emotional expressions is enhanced compared with that of neutral expressions in the match-to-sample paradigm.

Furthermore, as predicted, the results of Experiment 4 demonstrated that the conscious perception of normal-angry expressions was enhanced compared with that of anti-expressions, which had a comparable amount of featural distortions with normal-angry expressions. Likewise, the results of Experiment 5 indicated that the perception of angry expressions looking toward participants was more accurate than that for angry expressions looking away from participants, for identical photographs presented in different visual fields. These results are compatible with previous studies that have used the visual search paradigm, which found that the detection of normal-angry expressions was more rapid than that for anti-expressions^38–46^. However, these studies only measured the speed of behavioral responses and did not assess the subjective conscious awareness of stimuli. To our knowledge, the current results provide the first evidence that the conscious perceptual enhancement of emotional facial expressions could be attributed to their emotional characteristics, irrespective of their visual features.

The results of Experiments 1–3 and 5 showed that identification accuracy was higher for negative expressions than positive expressions, consistent with several previous studies that used different paradigms^9,12,13,18,19,21^. However, other studies reported no valence differences^11,15–17^ or an advantage for positive expressions^8,10,20^. Our results provide further support that conscious awareness of photographs of negative expressions is enhanced compared with that of positive expressions using the matching-to-sample paradigm.

The data of Experiments 1–3 did not clearly show main effects or interactions related to the visual field. We only found a nonsignificant tendency of the main effect, indicating a left visual field advantage, in the analysis of the data of Experiment 2, which only weakly suggests right hemispheric dominance. Two earlier studies that used schematic faces in the match-to-sample paradigm also reported inconsistent findings regarding hemispheric functional asymmetry; one reported general right hemisphere dominance^48^ and the other reported different patterns across emotional expressions^49^. Further, as was the case in the present study, other studies that tested the effect of visual field on the various processes of photographic emotional expressions reported null outcomes^44,63,64^. These findings appear to be in line with neurophysiological evidence that rapid activity in the amygdala^25^ and visual cortices^22^ in response to photographs of emotional expressions was not clearly lateralized. A neuroimaging study of a large sample (*n* = 108) was also unable to provide clear evidence of lateralization of activity in face-processing-related brain regions during the observation of facial photographs^65^. We speculate that the rapid processing of facial expressions in conscious awareness may not be clearly lateralized. However, the results of this study should be interpreted cautiously due to several limitations. First, our sample size was designed to detect the strong effect of emotional expression. Hence, we may have failed to detect a weak laterality effect. Second, although we used a secondary fixation task to ensure fixation^52^, a previous study has reported that a certain amount of eye movement occurred even with active fixation^66^. We did not use other fixation tasks and did not measure eye movements to confirm stimulation of the single hemisphere. Future studies testing larger samples and controlling for fixation may reveal hemispheric functional asymmetry in the conscious awareness of facial expressions.

Experiments 4 and 5 yielded two other interesting findings. First, in Experiment 4, the difference in identification accuracy between anti-angry and neutral expressions did not reach significance, although accuracy was higher for anti-angry expressions. In the anti-angry expressions, the facial features of angry expressions were changed by the same amount in the opposite direction to those of angry expressions relative to neutral expressions^47^; therefore, our results support the idea that visual features may not be critical for enhanced conscious awareness of angry expressions. However, the result should be interpreted with caution as it was in fact non-significant^67^. Our sample size also lacked the power to detect weak effects. Second, in Experiment 5, there was an effect of face direction, where identification accuracy was greater for neutral expressions looking away versus those looking toward the participants. Several previous studies have shown that direct gaze rapidly enhanced various types of cognitive processing^68,69^, which may have interfered with the conscious identification of faces. Alternatively, this result may be related to the negative connotations of gaze aversion^70,71^. It was pointed out that the influence of gaze could differ depending on the communication contexts^69^. Although our focus was on the effect of emotion, the modulatory effect of gaze direction on conscious awareness of faces could be an important target for future research.

Our findings have theoretical implications. First, our results indicate that the conscious processing of emotional facial expressions is modulated by emotional factors. There remains debate regarding whether the enhanced conscious perception of facial expressions is related to emotional or visual factors. It is unlikely that the visual factors are irrelevant, as some previous studies have revealed that the visual characteristics of emotional facial expressions (e.g. curved mouth) enhanced conscious perception of the expressions compared with neutral expressions^11,20,21^. However, the view that solely visual factors account for the enhanced conscious awareness of emotional facial expressions implies that the psychological mechanisms underlying emotional expression processing do not involve rapid interactions between visual and emotional processing. By contrast, data suggest that humans have psychological mechanisms by which emotional factors modulate the conscious visual processing of facial expressions, which corroborates the neuroscientific findings discussed below. Second, the present findings imply that the conscious awareness of external stimuli could be modulated as a function of emotion. There remains debate regarding the adaptive functions of conscious awareness, and some researchers speculate that conscious experience is an epiphenomenon without function^7^. By contrast, there is relatively little debate about the adaptive functions of emotion that prioritize the processing of biologically significant stimuli^1,2,72,73^. Although evidence suggests that physiological and behavioral emotional responses could arise before conscious awareness of stimuli^2,74,75^, conscious identification of the stimuli would facilitate adaptive responses. It was pointed out that conscious processing has various advantages, such as increasing flexibility in responses^4,5^. For example, a previous lesion study has reported that a blindsight patient could feel the familiarity in response to the unseen photograph of his wife but wrongly guessed her to be his friend^39^. In such cases, unconscious emotional responses should be refined with conscious identification of the stimuli. Taken together, our results suggest that one of the most important functions of conscious awareness is to prioritize the processing of emotionally significant stimuli, such as emotional facial expressions, in conscious experiences.

Our findings suggest that the neural mechanisms underlying emotional expression perception include the amygdala. Consistent with the results of our experiments, numerous previous neuroimaging studies have shown that the amygdala was more active in response to emotional (both angry and happy) expressions than neutral expressions^76,77^. In line with Experiment 4, a previous lesion study reported that amygdala damage impaired the rapid detection of normal-angry expressions compared with anti-angry expressions^44^. As in the case of Experiment 5, a previous neuroimaging study showed that images of angry expressions looking toward the participant elicited stronger amygdala activity than angry expressions looking away from the participant^61^. Furthermore, numerous neuroimaging^24,26,27^ and neurophysiological^25,28^ studies indicated that the amygdala could be preconsciously activated in response to emotional facial expressions, and that it could then modulate activity in the visual cortices during the conscious processing of facial expressions. Consistently, anatomical studies in animals indicated that the amygdala receives visual input via the subcortical visual pathway^78^, and that it sends direct projections to the visual cortices^79^. Based on ample neuroscientific evidence, some researchers theorized that the amygdala conducts emotional processing for facial expressions before the conscious awareness of faces and then modulates activity in the cortical visual areas related to the conscious perception of facial expressions^80,81^. However, as there is no direct evidence showing an association between the amygdala and enhanced conscious awareness of emotional facial expressions (cf. emotional words^82,83^), future neuroimaging or lesion studies are warranted.

### Limitations

In addition to the limitations of the methodology discussed above, including the small sample size and lack of strict fixation control, this study had several other limitations. First, we did not assess individual differences in the enhanced conscious awareness of facial expressions. As several previous studies have shown that the detection of facial expressions due to emotional factors could be modulated by personality traits, such as neuroticism^41^ and autistic traits^42^, such traits could modulate performance in the match-to-sample paradigm. Future studies should investigate this issue.

Second, we tested relatively few stimuli. Because we repeatedly presented photographs of three individuals in each experiment, the results may have reflected learning effects. Additionally, we tested only Caucasian and Japanese faces with angry, happy, and neutral expressions; whether the results generalize to other races and emotional expressions remains to be determined. Therefore, further studies should test additional facial expression stimuli.

Finally, we used photographs of facial expressions as stimuli, which may have lacked ecological validity. Because facial expressions in real life are dynamic^84^, conscious awareness of dynamic facial expressions needs to be tested. Several previous studies have suggested that psychological and neural processing is enhanced for dynamic emotional facial expressions compared with static emotional expressions^84^. For example, a previous psychological study reported that the detection of dynamic facial expressions was more efficient than that of static expressions in the visual search paradigm^85^. Functional neuroimaging studies reported that the observation of dynamic versus static facial expressions induced stronger activity in the visual cortices and amygdala^86,87^. These data suggest that conscious awareness of dynamic emotional expressions may be enhanced compared with that of static emotional expressions. Testing this idea using the present paradigm is an important target for future research.

## Conclusions

Five psychological experiments using photographs of facial expressions in the match-to-sample paradigm consistently revealed that the conscious identification of emotional facial expressions was more accurate than that for neutral facial expressions. Furthermore, Experiment 4, which compared normal- and anti-angry expressions, showed that this superiority could not be attributed to the physical features of emotional facial expressions. Experiment 5, which tested the interaction between emotional expression and face direction, further showed that the identification of angry expressions looking toward participants was more accurate than those looking away from participants, although the faces were physically the same. These results suggest that the conscious awareness of emotional facial expressions is enhanced by their emotional significance.

## Methods

### Experiments 1–3

#### Participants

In total, 18 (9 females and 9 males; mean ± *SD* age, 22.8 ± 2.2 years), 13 (7 females and 6 males; mean ± *SD* age, 20.3 ± 1.7 years), and 12 (4 females and 8 males; mean ± SD age, 20.2 ± 1.7 years) Japanese volunteers participated in Experiments 1 (Caucasian male face), 2 (Japanese male face), and 3 (Japanese female face), respectively. We estimated the sample size for each experiment via an a priori power analysis using G*Power^88^ on the basis of a within-subjects ANOVA with six measurements, an *α* level of 0.05, power of 0.80, and effect size *f* of 0.4 (strong). The effect size was estimated from the results of a previous psychological study^44^ that reported a detection advantage for emotional versus neutral expressions in the visual search paradigm with a large effect size (i.e. *d* = 1.03). The results showed that eight participants would be needed. All participants were right-handed and had normal or corrected-to-normal visual acuity. The participants received an explanation regarding the experimental procedure and provided written informed consent. This study was approved by Ethics Committee of the Primate Research Institute, Kyoto University, Japan, and conducted in accordance with the institutional ethical provisions and the Declaration of Helsinki.

#### Design

The experiment had a within-subjects two-factorial design, with emotion (anger, happiness, or neutral) and visual field (left or right) as the factors. We used the within-subjects design because it is generally more efficient than the between-subjects design, as it removes subject variance from error terms^89^. Our primary interest was the effect of emotion. The neutral expression was regarded as the control/baseline condition.

#### Stimuli

The target faces in Experiment 1 (Fig. 1B) were grayscale photographs of three males expressing anger, happiness, and neutral emotions chosen from a standardized facial expression set^62^. The target faces in Experiments 2 and 3 were color photographs of three males and three females (Fig. 1C), respectively, expressing anger, happiness, and neutral emotions. The photographs were selected from the ATR facial expression image Database (ATR-Promotions, Seika-cho, Japan). The faces were oval shaped for the purpose of minimizing extraneous clues (e.g. hair) and subtended a visual angle (i.e. the angle a viewed object subtends at the eye) of 7.0° vertical × 5.0° horizontal.

#### Apparatus

The experimental trials were controlled using SuperLab software (Cedrus, San Pedro, CA, USA) on a Windows computer. The participant observed a wide translucent screen (visual angle of 47° vertical × 63° horizontal) from a viewing distance of 0.57 m. The stimulus was backward projected from a liquid crystal projector (Impression 970, ASK, Sunnyvale, CA, USA).

#### Procedure

The experiments were conducted individually in a soundproof room. The participant was comfortably seated with their head supported by a chin-and-forehead rest. Figure 1A shows a representative illustration of the sequence of events in each trial. First, a lower-case letter flashed (200 ms) in the center of the screen. Then the target face was briefly exposed (100 ms) in either the left or right visual field (12 degrees peripherally from the center), and finally a test panel containing nine faces was presented. The participant’s first task was to report a letter in the center. This letter-reporting task was conducted to maintain fixation at the center of the screen^48^ and to make face perception more difficult^49^. The letter identification task accuracy was 100%. The participant’s second task, which was of interest to us, was to simply choose the perceived image from the nine choices via a verbal response. If the participant failed to perceive a face, he/she was asked to give up without guessing. The responses could be made in a leisurely fashion (i.e. this was not a reaction-time task).

There were 18 for each condition (anger-left, anger-right, happiness-left, happiness-right, neutral-left, and neutral-right), for a total of 108 trials per participant. To avoid fatigue and drowsiness, the participants took a short rest after finishing 36 trials. They completed a block of 10 training trials to familiarize themselves with the study procedure.

#### Data analysis

All statistical tests were performed using SPSS 16.0 J software (SPSS Japan, Tokyo, Japan). The mean percentage of correct responses (number of correct trials/ total trials for each condition × 100) was calculated for each condition and participant in each experiment. Then these were analyzed using a two-way repeated-measures ANOVA with emotion (angry, happy, and neutral) and visual field (left and right) as factors. Multiple comparisons were conducted using the Ryan method. The *α*-level for all analyses was set to 0.05. The assumption of sphericity was met for all experiments (Mauchly’s *W* > 0.8, *p* > 0.239). Preliminary analyses showed no significant main effects or interactions related to participant sex (*p* > 0.10); hence, we reported the results without this factor.

### Experiment 4

#### Participants

Eighteen Japanese volunteers (6 females and 12 males; mean ± SD age, 21.1 ± 2.2 years) participated in this experiment. The sample size was determined based on the a priori power analysis in Experiment 1. All participants were right-handed and had normal or corrected-to-normal visual acuity. The participants gave written informed consent.

#### Design

The experiment was identical to that of Experiment 1 except that the levels of the emotion factor were normal-anger, anti-anger, and neutral.

#### Stimuli

Images of three males showing angry and neutral emotional expressions were chosen from the aforementioned standard set^62^ (Fig. 1D). To make anti-expressions, we used computer-morphing software (Foolproof Utilities for Facial Image Manipulation, ATR) on a Linux computer. Using the facial images showing angry and neutral expressions (the latter was included as a norm), we created faces with artificial expressions by changing the positions of 79 feature points on the angry faces. After calculating the differences between the feature points on the angry and neutral faces, we were able to determine the positions of the new feature points by moving each point by the same amount in the opposite direction from that in the angry face. This manipulation made it possible to create faces for which the parts had a comparable degree of distinctiveness to that of the angry faces, but with the emotional load effectively filtered out due to their artificiality (Fig. 1D). The faces were oval shaped.

To verify the emotional neutrality of the anti-expressions, we preliminarily showed all stimuli in this experiment to 13 Japanese volunteers (7 females and 6 males; mean ± *SD* age, 21.1 ± 1.2 years) who did not take part in the main experiment. The participants evaluated the emotional valence of each stimulus using a 9-point scale ranging from − 4 (negative) to + 4 (positive). The experiments were conducted individually with the same apparatus used in the main experiment and the participants’ ratings were recorded using a keyboard. The ratings indicated that anti-expressions were perceived as emotionally neutral (mean ± *SD,* − 0.3 ± 0.3). Then, we conducted a repeated-measures ANOVA with emotion as a factor. The main effect of emotion was significant (*F*(2,24) = 106.6, *p* = 0.000, *η*^2^_*p*_ = 0.90). Multiple comparisons using the Ryan method revealed that the differences between the normal- and anti-angry expressions and between the normal-angry and neutral expressions were both significant (*t*(24) > 12.5, *p* < 0.001), whereas the difference between anti-angry and neutral expressions was not significant (*t*(24) = 0.1, *p* = 0.890).

#### Procedure

The procedure was identical to that of Experiment 1.

#### Data analysis

The data analysis was identical to that of Experiment 1, except that the levels of the emotion factor were normal-anger, anti-anger, and neutral. The assumption of sphericity was confirmed for the data of all experiments (Mauchly’s *W* > 0.8, *p* > 0.411).

### Experiment 5

#### Participants

Thirty-five Japanese volunteers (12 females and 23 males; mean ± *SD* age, 20.1 ± 0.9 years) participated in this experiment. The sample size was determined via an a priori power analysis using G*Power^88^. We assumed a within-subjects ANOVA to test the simple-simple main effect of face direction with two measurements and an *α* level of 0.05, power of 0.80, and effect size *f* of 0.25 (medium). The effect size was estimated from the results of a previous neuroimaging study^61^ that reported higher activity in the amygdala in response to angry expressions looking toward versus away from participants with a medium effect size (i.e. *d* = 0.50). The results showed that 34 participants would be needed. All participants were right-handed and had normal or corrected-to-normal visual acuity. The participants provided written informed consent.

#### Design

The experiment had a within-subjects three-factorial design, with emotion (anger, happiness, and neutral), face direction (toward, and away), and face angle (0°, 15°, 30°, and 45°) as factors.

#### Stimuli

The target stimuli were color photographs of three males expressing anger, happiness, and neutral emotions at angles of 0°, 15°, 30°, and 45° from the left (Fig. 1E). The images were selected from the ATR Facial Expression Image Database (ATR-Promotions, Seika-cho, Japan). The models depicted in all of the stimuli were looking forward. Mirror images of all of the stimuli were also prepared. These faces were oval shaped and subtended a visual angle of 7.0° vertical × 5.0° horizontal. Preliminary tests with naïve participants indicated that faces looking toward the participants at angles of 15° and 30° produced the impression of eye contact in the current experimental environment. Test panels contained nine images of three individuals showing three different expressions at the same face direction and angle as the target faces.

#### Procedure

The procedure was identical to that of Experiment 1 except that there were six presentations for each of the 24 conditions (anger-toward-0°, anger-toward-15°, anger-toward-30°, anger-toward-45°, anger-away-0°, anger-away-15°, anger-away-30°, anger-away-45°, happiness-toward-0°, happiness-toward-15°, happiness-toward-30°, happiness-toward-45°, happiness-away-0°, happiness-away-15°, happiness-away-30°, happiness-away-45°, neutral-toward-0°, neutral-toward-15°, neutral-toward-30°, neutral-toward-45°, neutral-away-0°, neutral-away-15°, neutral-away-30°, neutral-away-45°), thus making a total of 144 trials for each participant.

#### Data analysis

The data analysis was identical to that of Experiment 1, except that the within-subjects factors were emotion, face angle, and face direction. The assumption of sphericity was not met for the main effect of angle (Mauchly’s *W* = 0.7, *p* = 0.043); therefore, the significance of this factor was evaluated using Huynh–Feldt-adjusted degrees of freedom.

### Supplementary Information

Supplementary Table S1.

## Data Availability

All data analyzed during this study are included in this published article and its Supplementary Information files.
