# Supplementary material for: Influence of stimulus manipulation on conscious awareness of emotional facial expressions in the match-to-sample paradigm
Source: Sci Rep. 2023 Nov 25;13:20727. doi: 10.1038/s41598-023-47995-9 (PMC10676436; doi:10.1038/s41598-023-47995-9)
Supplement: Supplementary file 1 — Supplementary Table S1. [file 41598_2023_47995_MOESM1_ESM.pdf]

Supplementary Table S1. The percentage of correct identification for each condition in each participant.

Experiment 1

ID	Age	Sex	Left visual field			Right visual field		
			AN	HA	NE	AN	HA	NE
1	26	M	61.1	33.3	11.1	44.4	38.9	22.2
2	23	F	77.8	55.6	22.2	72.2	61.1	27.8
3	23	F	77.8	50.0	16.7	88.9	66.7	27.8
4	23	M	72.2	33.3	27.8	66.7	50.0	50.0
5	23	M	88.9	50.0	38.9	77.8	50.0	5.6
6	27	F	94.4	61.1	16.7	61.1	61.1	33.3
7	24	M	50.0	33.3	16.7	83.3	55.6	27.8
8	21	F	77.8	50.0	5.6	77.8	44.4	22.2
9	20	F	77.8	66.7	33.3	66.7	38.9	0.0
10	20	F	88.9	66.7	66.7	83.3	77.8	44.4
11	20	F	72.2	88.9	27.8	55.6	61.1	22.2
12	20	F	83.3	27.8	11.1	33.3	44.4	27.8
13	21	M	77.8	44.4	16.7	77.8	55.6	38.9
14	21	M	61.1	44.4	5.6	72.2	38.9	5.6
15	24	F	50.0	33.3	27.8	50.0	38.9	5.6
16	21	M	77.8	55.6	16.7	83.3	77.8	38.9
17	20	M	66.7	55.6	44.4	55.6	44.4	22.2
18	25	M	77.8	50.0	33.3	61.1	66.7	11.1

Experiment 2

ID	Age	Sex	Left visual field			Right visual field		
			AN	HA	NE	AN	HA	NE
1	25	F	66.7	44.4	16.7	66.7	50.0	22.2
2	18	M	77.8	72.2	50.0	61.1	61.1	44.4
3	18	F	44.4	11.1	33.3	44.4	44.4	11.1
4	19	M	61.1	50.0	0.0	55.6	61.1	33.3
5	21	M	61.1	50.0	50.0	38.9	33.3	22.2
6	19	F	77.8	44.4	11.1	61.1	38.9	33.3
7	18	M	88.9	55.6	44.4	77.8	44.4	22.2
8	21	F	44.4	44.4	33.3	27.8	11.1	5.6

9	23	F	27.8	55.6	27.8	44.4	33.3	16.7
10	20	F	44.4	44.4	5.6	27.8	38.9	22.2
11	22	M	61.1	44.4	11.1	27.8	22.2	16.7
12	22	F	50.0	22.2	16.7	50.0	33.3	16.7
13	20	M	50.0	50.0	44.4	33.3	38.9	11.1

Experiment 3

ID	Age	Sex	Left visual field			Right visual field		
			AN	HA	NE	AN	HA	NE
1	20	F	50.0	11.1	16.7	27.8	16.7	5.6
2	20	F	66.7	38.9	11.1	33.3	33.3	27.8
3	19	M	72.2	50.0	33.3	55.6	22.2	22.2
4	20	M	50.0	44.4	22.2	44.4	38.9	16.7
5	19	M	72.2	66.7	61.1	72.2	27.8	38.9
6	21	M	77.8	88.9	72.2	94.4	77.8	50.0
7	21	F	61.1	55.6	38.9	66.7	50.0	22.2
8	20	M	83.3	72.2	22.2	77.8	77.8	33.3
9	19	F	72.2	77.8	72.2	77.8	77.8	50.0
10	25	M	72.2	38.9	33.3	77.8	66.7	38.9
11	19	M	66.7	27.8	27.8	88.9	66.7	55.6
12	19	M	94.4	77.8	66.7	77.8	83.3	38.9

Experiment 4

ID	Age	Sex	Left visual field			Right visual field		
			AN	Anti	NE	AN	Anti	NE
1	19	M	22.2	16.7	22.2	11.1	27.8	22.2
2	20	F	38.9	38.9	0.0	33.3	11.1	11.1
3	21	F	61.1	38.9	16.7	27.8	0.0	11.1
4	21	M	55.6	5.6	33.3	44.4	16.7	27.8
5	20	M	55.6	16.7	22.2	11.1	16.7	33.3
6	20	F	33.3	0.0	16.7	44.4	5.6	16.7
7	20	M	33.3	16.7	16.7	38.9	0.0	5.6
8	19	F	72.2	55.6	11.1	72.2	61.1	16.7
9	21	F	61.1	72.2	27.8	72.2	66.7	22.2

10	20	F	66.7	38.9	33.3	38.9	44.4	50.0
11	29	F	22.2	27.8	33.3	50.0	11.1	0.0
12	20	F	38.9	11.1	16.7	38.9	11.1	16.7
13	22	M	55.6	33.3	50.0	55.6	27.8	38.9
14	22	M	27.8	22.2	16.7	33.3	16.7	22.2
15	21	F	50.0	33.3	5.6	55.6	27.8	22.2
16	21	M	22.2	33.3	16.7	55.6	16.7	33.3
17	21	M	50.0	50.0	33.3	55.6	16.7	61.1
18	22	M	16.7	27.8	27.8	22.2	44.4	5.6

Experiment 5

ID	Age	Sex	0°						15°						30°						45°					
			Toward			Away			Toward			Away			Toward			Away			Toward			Away		
			AN	HA	NE	AN	HA	NE	AN	HA	NE	AN	HA	NE	AN	HA	NE	AN	HA	NE	AN	HA	NE	AN	HA	NE
1	21	M	66.7	50.0	0.0	66.7	66.7	0.0	66.7	66.7	0.0	66.7	50.0	0.0	50.0	33.3	0.0	50.0	83.3	16.7	16.7	16.7	0.0	66.7	33.3	33.3
2	22	M	33.3	16.7	16.7	50.0	16.7	33.3	33.3	16.7	0.0	50.0	33.3	16.7	16.7	33.3	33.3	50.0	33.3	16.7	16.7	33.3	33.3	50.0	50.0	33.3
3	21	F	33.3	16.7	33.3	33.3	0.0	0.0	16.7	0.0	16.7	0.0	0.0	0.0	33.3	16.7	33.3	0.0	0.0	50.0	33.3	50.0	0.0	16.7	33.3	0.0
4	20	F	33.3	50.0	16.7	33.3	16.7	16.7	33.3	33.3	16.7	33.3	50.0	0.0	33.3	66.7	0.0	0.0	33.3	0.0	33.3	50.0	16.7	50.0	50.0	33.3
5	19	M	0.0	66.7	16.7	33.3	33.3	0.0	33.3	66.7	50.0	33.3	66.7	0.0	66.7	16.7	16.7	16.7	50.0	16.7	50.0	66.7	16.7	16.7	66.7	66.7
6	20	F	33.3	33.3	0.0	16.7	33.3	33.3	66.7	33.3	16.7	16.7	50.0	0.0	66.7	33.3	0.0	33.3	33.3	50.0	50.0	50.0	16.7	16.7	33.3	33.3
7	19	F	50.0	16.7	0.0	50.0	33.3	16.7	50.0	33.3	0.0	50.0	33.3	0.0	33.3	16.7	0.0	83.3	50.0	0.0	33.3	50.0	33.3	16.7	33.3	50.0
8	19	M	33.3	0.0	50.0	33.3	16.7	0.0	16.7	33.3	33.3	50.0	16.7	0.0	33.3	66.7	0.0	33.3	33.3	33.3	16.7	50.0	16.7	33.3	16.7	50.0
9	21	M	33.3	33.3	16.7	33.3	33.3	33.3	0.0	50.0	16.7	16.7	50.0	33.3	33.3	16.7	16.7	16.7	50.0	33.3	33.3	33.3	50.0	33.3	16.7	33.3
10	19	M	16.7	66.7	33.3	33.3	33.3	0.0	50.0	50.0	50.0	33.3	16.7	16.7	16.7	33.3	33.3	0.0	0.0	33.3	0.0	16.7	33.3	0.0	16.7	0.0
11	19	M	16.7	16.7	0.0	50.0	33.3	0.0	33.3	33.3	33.3	16.7	16.7	33.3	83.3	16.7	16.7	16.7	16.7	0.0	33.3	33.3	66.7	16.7	33.3	16.7
12	20	M	50.0	33.3	0.0	33.3	33.3	16.7	16.7	50.0	33.3	50.0	33.3	16.7	16.7	33.3	16.7	33.3	33.3	16.7	16.7	0.0	33.3	16.7	33.3	16.7
13	20	M	33.3	33.3	16.7	66.7	33.3	0.0	16.7	50.0	16.7	66.7	50.0	33.3	50.0	50.0	16.7	50.0	66.7	0.0	50.0	33.3	0.0	16.7	50.0	16.7
14	21	M	33.3	50.0	33.3	50.0	33.3	0.0	66.7	66.7	50.0	0.0	83.3	33.3	16.7	66.7	0.0	16.7	50.0	50.0	50.0	50.0	33.3	16.7	16.7	66.7
15	21	M	50.0	33.3	0.0	66.7	50.0	33.3	50.0	33.3	16.7	16.7	33.3	33.3	50.0	33.3	0.0	33.3	16.7	0.0	33.3	0.0	0.0	50.0	50.0	16.7
16	20	F	50.0	33.3	0.0	50.0	50.0	16.7	50.0	33.3	16.7	33.3	33.3	16.7	50.0	33.3	16.7	33.3	50.0	0.0	16.7	66.7	33.3	50.0	33.3	33.3
17	20	F	50.0	16.7	0.0	33.3	0.0	0.0	0.0	0.0	0.0	0.0	16.7	16.7	33.3	0.0	0.0	16.7	0.0	0.0	33.3	16.7	33.3	0.0	16.7	0.0
18	21	M	16.7	16.7	0.0	33.3	16.7	33.3	66.7	16.7	16.7	33.3	16.7	16.7	0.0	16.7	16.7	33.3	33.3	16.7	50.0	33.3	33.3	33.3	0.0	0.0
19	21	F	50.0	33.3	16.7	50.0	16.7	33.3	66.7	16.7	0.0	83.3	16.7	0.0	33.3	0.0	0.0	33.3	16.7	16.7	33.3	0.0	0.0	16.7	33.3	16.7
20	19	F	66.7	16.7	0.0	33.3	16.7	33.3	33.3	0.0	0.0	33.3	50.0	16.7	50.0	0.0	0.0	33.3	33.3	16.7	16.7	16.7	50.0	33.3	33.3	16.7
21	21	M	33.3	33.3	0.0	16.7	0.0	0.0	33.3	33.3	16.7	16.7	33.3	0.0	33.3	33.3	0.0	50.0	16.7	16.7	33.3	16.7	16.7	16.7	0.0	0.0

22	20	M	66.7	33.3	0.0	33.3	33.3	0.0	33.3	0.0	16.7	50.0	16.7	0.0	66.7	50.0	0.0	50.0	0.0	0.0	50.0	66.7	16.7	66.7	66.7	50.0
23	20	F	33.3	33.3	66.7	33.3	66.7	16.7	50.0	33.3	16.7	33.3	33.3	33.3	66.7	33.3	16.7	16.7	33.3	33.3	16.7	50.0	33.3	33.3	33.3	16.7
24	19	M	33.3	33.3	33.3	33.3	66.7	16.7	33.3	16.7	0.0	0.0	33.3	16.7	50.0	33.3	0.0	66.7	50.0	16.7	16.7	33.3	33.3	16.7	16.7	50.0
25	21	M	50.0	50.0	33.3	33.3	33.3	16.7	50.0	33.3	16.7	16.7	50.0	16.7	66.7	50.0	16.7	50.0	50.0	33.3	50.0	33.3	66.7	66.7	50.0	66.7
26	20	M	16.7	66.7	50.0	16.7	66.7	33.3	66.7	33.3	33.3	33.3	16.7	33.3	50.0	83.3	16.7	33.3	66.7	33.3	66.7	33.3	33.3	33.3	16.7	33.3
27	19	M	33.3	33.3	16.7	66.7	33.3	0.0	50.0	50.0	16.7	33.3	0.0	16.7	66.7	50.0	0.0	33.3	66.7	16.7	16.7	33.3	16.7	50.0	16.7	0.0
28	20	M	50.0	16.7	16.7	33.3	0.0	16.7	33.3	16.7	33.3	50.0	33.3	16.7	33.3	33.3	0.0	0.0	33.3	16.7	33.3	16.7	16.7	33.3	33.3	16.7
29	21	F	16.7	33.3	33.3	33.3	16.7	50.0	33.3	50.0	16.7	0.0	50.0	50.0	33.3	66.7	33.3	16.7	16.7	50.0	33.3	50.0	33.3	16.7	16.7	33.3
30	22	M	33.3	0.0	16.7	0.0	16.7	0.0	16.7	0.0	16.7	0.0	0.0	0.0	16.7	0.0	0.0	0.0	16.7	16.7	16.7	0.0	16.7	16.7	16.7	0.0
31	19	F	16.7	33.3	0.0	83.3	33.3	33.3	50.0	50.0	0.0	33.3	16.7	16.7	50.0	16.7	16.7	33.3	50.0	0.0	33.3	0.0	16.7	50.0	33.3	16.7
32	20	F	33.3	16.7	0.0	66.7	16.7	16.7	33.3	16.7	16.7	33.3	33.3	33.3	16.7	16.7	33.3	50.0	33.3	16.7	50.0	16.7	16.7	16.7	16.7	0.0
33	19	F	33.3	33.3	0.0	33.3	50.0	0.0	50.0	16.7	0.0	33.3	50.0	0.0	33.3	16.7	0.0	50.0	50.0	16.7	33.3	33.3	0.0	16.7	33.3	16.7
34	19	M	66.7	33.3	33.3	50.0	33.3	50.0	50.0	16.7	16.7	66.7	33.3	33.3	83.3	16.7	0.0	50.0	33.3	50.0	33.3	16.7	50.0	50.0	33.3	16.7
35	19	M	50.0	33.3	33.3	16.7	50.0	0.0	66.7	0.0	16.7	33.3	0.0	16.7	50.0	33.3	16.7	50.0	0.0	16.7	33.3	33.3	33.3	0.0	0.0	0.0

F = Female; M = Male; AN = Anger; HA = Happiness; NE = Neutral.
